# A digestive cartridge reduces intestinal injury in a murine model of necrotizing enterocolitis

**DOI:** 10.1371/journal.pone.0348200

**Published:** 2026-04-30

**Authors:** Sarah Z. Wang, Djanira Fernandes, Savas T. Tsikis, Thomas I. Hirsch, Amy Pan, Mikayla Quigley, Valeria Ruiz, Scott C. Fligor, Carter R. Petty, Greta Loring, Stephen Davia, Juhye Kang, Mark Puder

**Affiliations:** 1 Department of Surgery, Vascular Biology Program, Boston Children’s Hospital, Boston, Massachusetts, United States of America; 2 Harvard Medical School, Boston, Massachusetts, United States of America; 3 Biostatistics and Research Design Center, Boston Children’s Hospital, Boston, Massachusetts, United States of America; 4 Alcresta Therapeutics, Waltham, Boston, Massachusetts, United States of America; Kobe University Graduate School of Medicine School of Medicine, JAPAN

## Abstract

**Background:**

Formula feeding is associated with necrotizing enterocolitis (NEC), a life-threatening gastrointestinal illness affecting premature infants. The presence of undigested fat in enteral formula may mediate NEC severity. The immobilized lipase cartridge (ILC) is an FDA-cleared digestive device that hydrolyzes triglyceride fat in formula into readily absorbable free fatty acids and monoglycerides. We hypothesized that use of a new ILC prototype designed to support increased feeding frequency in infants will reduce NEC mortality and disease severity in a murine model.

**Methods:**

NEC was induced in C57BL/6J mice from post-natal day 4–6 via oral gavage of lipopolysaccharide-containing formula 4x/day and hypoxia (5% O_2_) exposure 2x/day. Littermates were randomized to one of five groups: dam-fed (normal controls), ILC-digested formula, placebo-processed formula, NEC + ILC-digested formula, NEC+placebo-processed formula. Weights and mortality were measured daily. Mice were euthanized on P7 for assessment of NEC severity.

**Results:**

Normal controls had better survival rates than formula-fed and NEC mice. The NEC + ILC group exhibited lower clinical and histologic severity scores compared to the NEC+placebo group on masked evaluation.

**Conclusion:**

The administration of formula pre-digested by the ILC improved clinical sickness, gut appearance, and histologic severity of NEC compared to placebo. These findings support further investigation of the ILC as a non-invasive, preventive therapy for NEC in humans.

## Introduction

Formula feeding is associated with an increased incidence of necrotizing enterocolitis (NEC), a life-threatening gastrointestinal condition primarily affecting premature infants [1]. NEC leads to the loss of functional intestine and is the most common cause of pediatric short bowel syndrome [2]. While multiple meta-analyses have identified breastfeeding as a protective factor for reducing the incidence of NEC, breastmilk is not universally available [3,4]. Barriers to breastfeeding may include issues with lactation, inadequate workplace facilities, and limited social support [5]. Despite growing usage nationwide, donor breastmilk remains unavailable at 13.0% of U.S. hospitals with neonatal intensive care units (NICU) according to the Centers for Disease Control and Prevention (CDC) [6,7]. Additionally, donor breastmilk requires pasteurization which inactivates the lipase enzyme and may impact the bioavailability of various growth factors. Commercial infant formula fills an important existing gap; however, formula lacks the protective factors found in human breastmilk such as endogenous lipases that aid in fat digestion [8]. This is particularly important for premature neonates who are relatively lipase-deficient, resulting in impaired fat digestion [9]. Prior studies have demonstrated that digestive enzyme replacement can improve growth in infants and children with lipase deficiency due to exocrine pancreatic insufficiency [10,11].

The immobilized lipase cartridge (ILC) is an *ex vivo* enzymatic device designed to connect in-line with enteral feeding systems [12]. The ILC hydrolyzes triglyceride fat in enteral formula into readily absorbable free fatty acids and monoglycerides prior to reaching the patient. The immobilized lipase is retained in the cartridge and does not enter the body. In preclinical studies involving piglets with short bowel syndrome, use of the ILC increased the absorption of fat and fat-soluble vitamins, and reduced parenteral nutrition dependence [13,14]. Human studies have previously demonstrated the safety, tolerability, and effectiveness of the ILC in increasing plasma fatty acid levels [12,15]. Currently, the ILC is cleared by the U.S. Food and Drug Administration (FDA) for neonates, infants, and adult patients to hydrolyze fats during enteral feeding. A smaller, neonatal version of the ILC was utilized in this study, with a specific focus on meeting the nutritional and feeding frequency needs of neonates. This prototype contains the same immobilized lipase as the commercially available device.

The ILC has not previously been studied for the management of NEC. Previous research has demonstrated that infant formula containing partially digested fat in the form of free fatty acids and monoglycerides contributed to reduced disease severity in a murine model of NEC [16]. Specifically, Sodhi et al. concluded that mice fed “standard fat” formula developed more severe NEC phenotype compared to animals fed “predigested fat” or “very-low fat” formula. These investigators concluded that the intraluminal accumulation of undigested fat increases intestinal inflammation and proposed that the administration of formula containing pre-digested fat may overcome the relative lipase deficiency of the premature gut. It is important to recognize, however, that the pathogenesis of NEC is multifactorial. Multiple non-fat components, such as growth factors and antibodies, differ between infant formulas and breast milk. While these components may mediate NEC pathogenesis, our study sought to isolate the effect of the pre-digestion of enteral fat on NEC severity specifically. We hypothesized that use of a new, neonatal ILC prototype will reduce mortality and disease severity in a murine model of NEC compared to placebo.

## Methods

### Induction of necrotizing enterocolitis

**Fig 1:**
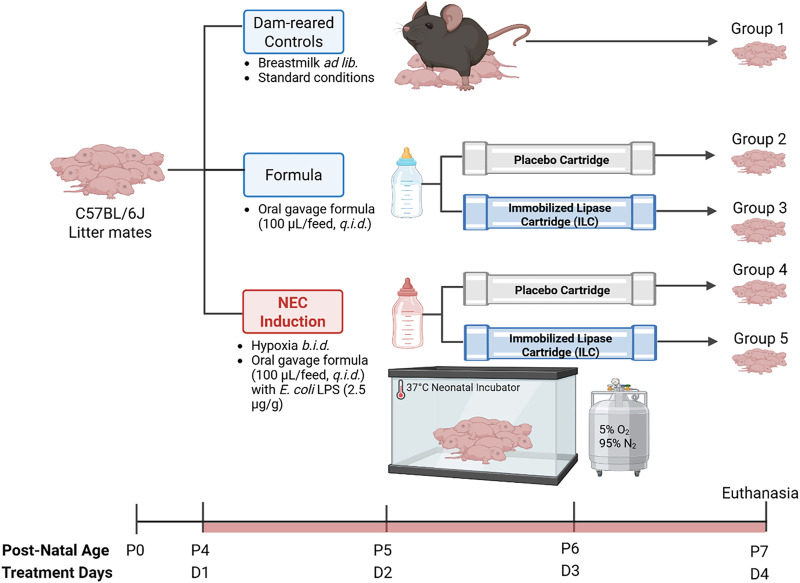
Experimental methodology investigating the effects of enteral formula pre-digested by an immobilized lipase cartridge (ILC) in a murine model of necrotizing enterocolitis (NEC). Group 1: dam-reared normal controls. Groups 2-5 are separated from the dams and formula fed four times per day for three days from post-natal day 4 (P4) through P7. Groups 2 and 3 are not subject to NEC, and receive formula processed through a placebo cartridge (group 2) or ILC (group 3). Groups 4 and 5 undergo experimental induction of NEC via a combination of feeding with formula containing *E. coli*-derived lipopolysaccharide (2.5 µg/g body weight) and twice-daily hypoxia exposure; group 4 and 5 receive formula processed through a placebo or ILC cartridge, respectively. *Ad lib –* ad libitum; *q.i.d.* – four times per day; *b.i.d.* – twice per day. Reprinted from [Biorender] under a CC BY license, with permission from [Biorender], original copyright [2026].

All protocols were approved by the Boston Children’s Hospital Institutional Animal Care and Use Committee (IACUC) according to the National Institutes of Health (NIH) Animal Research Advisory Committee and Animal Research: Reporting of In Vivo Experiments (ARRIVE) guidelines. The experimental methodology is summarized in **Fig 1**. Eight-week-old C57BL/6J mice (Jackson Laboratory, Bar Harbor, ME) were bred for several generations to ensure acclimatization to the facilities. Experimental NEC was induced in neonatal mice for three days from post-natal day 4 (P4) through P7 according to a previously published protocol, with the modification that purified *Escherichia coli* O127:B8 lipopolysaccharide (LPS) was used to induce NEC instead of enteric bacterial stock plus LPS [17].

On P4, littermate pups were randomized to one of five experimental groups: normal controls (maintained with the dam with *ad libitum* access to breastmilk), ILC-digested formula (formula + ILC), placebo cartridge-processed formula (formula + placebo), NEC + ILC-digested formula (NEC + ILC), and NEC + placebo cartridge-processed formula (NEC + placebo). Formula-fed pups were separated from the dam, housed in a 37°C infant incubator (GE Ohmeda Ohio Care Plus Incubator, Chicago, IL), and underwent oral gavage four times daily (100 µL/feed) from P4 through P6 at regular intervals over a 12-hour period using a 1.9 Fr single-lumen silicone catheter (Utah Medical Products Inc., Midvale, UT). Formula-fed pups did not have access to any other source of nutrition. The base enteral formula consisted of a 2:1 by-volume mixture of Similac Advance OptiGro (Abbott, Columbus, OH) and Esbilac Puppy Milk Replacer (Pet-Ag Inc., Hampshire, IL).

### Digestive cartridge and formula preparation

**Fig 2:**
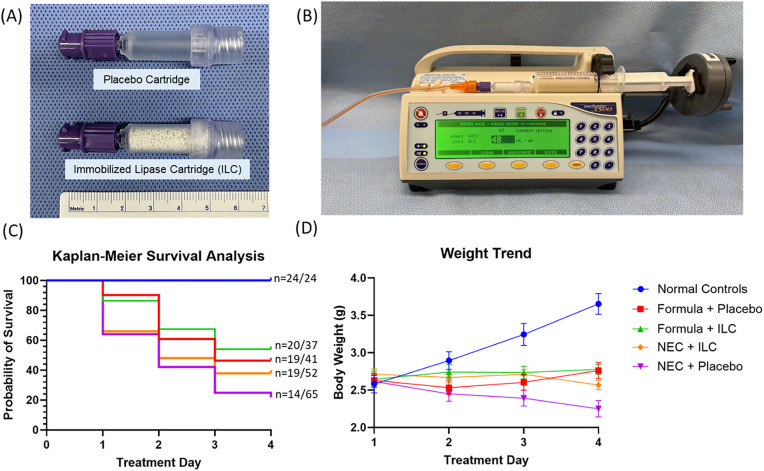
Survival analysis and weight trends. (A) Placebo and immobilized lipase cartridges; (B) Pump set up connecting the dispensing straw, digestive cartridge, and syringe containing formula (left to right); (C) Survival analysis with numbers demonstrating the ratio of survivors on D4 compared to initial cohort on D1; and (D) Weight trends of various experimental groups in a murine model of necrotizing enterocolitis (mean ± standard error of the mean). There were no significant differences in weight or survival at the end of the study between the NEC + ILC and NEC + placebo or Formula + ILC and Formula + placebo groups.

Immobilized lipase cartridges and placebo cartridges were prepared by Alcresta Therapeutics (Waltham, MA). The ILC consists of lipase enzyme covalently bonded to small beads (iLipase), with embedded filters on both ends for bead retention (Fig 2A). Placebo cartridges lacked the lipase beads but were otherwise identical in composition and structure. Cartridges were individually packaged for single use only. A new cartridge was used to process formula immediately before each feed.

For formula preparation, a prototype ILC or placebo was connected to an enteral syringe and an enteral feeding straw. The cartridge was primed by drawing the formula volume up through the straw, cartridge and into the syringe. The formula was then processed through the cartridge via a standard enteral feeding syringe pump (Smiths Medical, St. Paul, MN) at a controlled flow rate of 48 mL/hr under ambient conditions immediately prior to each feed (Fig 2B). Following exposure of formula to the ILC or placebo, the formula was mixed with *E. coli*-derived LPS (2.5 µg/g body weight, Sigma-Aldrich, St. Louis, MO). To complete NEC induction, animals were exposed to twice-daily hypoxia for 10 minutes after the first and third feed of each treatment day (5% O_2_/95% N_2,_ BioSpherix Ltd., Parish, NY). Daily weights and mortality rates from the initial sample sizes were assessed for each group (Figs 2C/D). On D4, animals were euthanized via decapitation for intestinal assessment and tissue collection.

### Assessment of clinical and histologic severity of NEC

**Fig 3:**
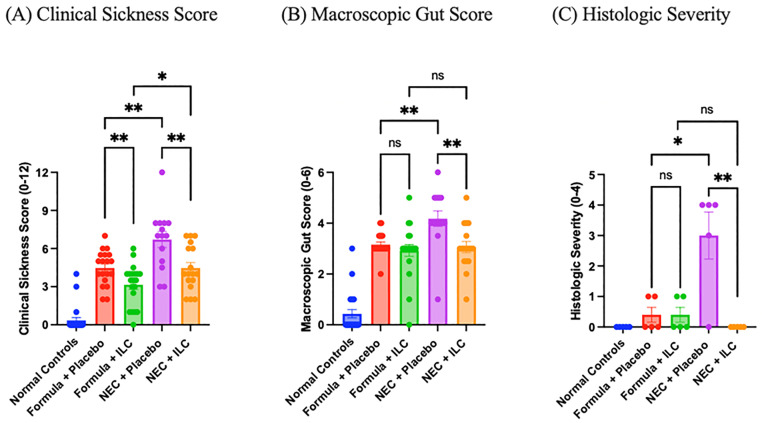
Clinical and histologic in a murine model of necrotizing enterocolitis as assessed by masked evaluators. (A) Clinical sickness score ranging from 0 to 12, and (B) Macroscopic gut score ranging from 0 to 6 were assessed by two evaluators; each point represents an individual animal’s score, calculated as the simple mean of the two scorers. (C) Histologic grade of NEC severity ranging from 0 to 4 as assessed by a certified veterinary pathologist. Values are expressed as mean ± standard error of the mean (SEM). *ns* indicates no significance; P > 0.05, * P ≤ 0.05, ** P ≤ 0.01.

On D4, two masked evaluators independently assessed clinical sickness (0–12, increasing severity) immediately before euthanasia and macroscopic gut appearance scores (0–6, increasing severity) immediately after euthanasia according to published protocol, using variables such as response to touch and gut friability, respectively (Fig 3A/B) [18]. The terminal ileum was resected, formalin-fixed (24 hours), paraffin-embedded, and stained with hematoxylin and eosin (H&E). A masked veterinary pathologist assessed the histologic severity of NEC using an established scoring system where NEC severity was graded on a scale from 0 to 4, with 4 representing the most severe histological findings (Fig 3C) [19].

### Humane endpoints

Humane endpoints were not used in this investigation given the inherent severity of the NEC model, in which weight loss/dehydration, discomfort, and mortality are expected outcomes of the disease. Number of animals used and mortalities are reported in Fig 2C. Every measure was taken to maximize animal welfare where appropriate in consultation with the Boston Children’s Hospital Institutional Animal Care and Use Committee (IACUC). Boston Children’s Hospital IACUC provided written consent for this study. The institutional animal ethics committee, including the lead veterinarian, specifically reviewed and approved the anticipated mortality in the present study design. Animals were examined a minimum of four times per day throughout the three-day experiment, with immediate additional monitoring and assistance available from study personnel and veterinary technicians. Monitoring was logged in written form and compliance with adherence to the study design was checked daily by veterinarian technicians. In this particular NEC model, pain or distress may result from handling, orogastric gavage, exposure to hypoxia and toxin (LPS), and maternal separation. To reduce animal stress from transfers and human investigators, all major procedures (i.e., gavage, general handling, hypoxia exposure) were limited to a single designated room in which this was the only active experiment. Animals were only removed from this area for planned euthanasia at the conclusion of the experiment. Handling and orogastric gavage of pups were performed only by trained personnel who have been individually observed and approved by the IACUC lead veterinarian. A contingency plan was created to immediately euthanize any animal that exhibited altered respiratory patterns or significant abdominal bloating suggestive of bowel perforation post-gavage; however, these signs were not observed. Hypoxia exposure was performed only by IACUC-approved study personnel. Animals that exhibited signs such as lethargy or altered respiratory pattern after hypoxia exposure were individually monitored, warmed, and manually stimulated until sternal (i.e., independently reactive). A contingency plan was created to immediately euthanize any animal that did not return to regular breathing patterns after manual stimulation; however, these signs were not observed. Maternal separation could cause distress in neonatal pups. Pups must be separated from dams so as to prevent access to breastmilk as part of the NEC model. Male adult mice could not be introduced as proxy companion animals as they could exhibit physical aggression towards pups. No anesthesia and/or analgesia exists to alleviate stress from maternal separation. However, pups were maintained in a warmed infant incubator with soft bedding to allow maintenance of normal body temperatures.

### Formula digestion and fatty acid analysis

Fat digestion (percentage hydrolysis) of the formula fats achieved by the ILC prototype cartridge was quantified using a commercially available assay for free fatty acids. Three separate lots of the ILC prototypes and formula mixture were tested under experimental conditions in order to measure the degree of fat hydrolysis. The percent hydrolysis through the placebo cartridges was also measured under identical conditions.

Complete fatty acid analysis of unprocessed formula, ILC-digested formula, and placebo cartridge-processed formula was conducted by OmegaQuant (Sioux Falls, SD) using gas chromatography with flame ionization detection. Placebo cartridges without beads (empty cartridges) and placebo cartridges containing inactive beads (lacking the lipase enzyme) were utilized. The fat concentration in the formula was calculated as the sum of all individual fatty acids measured, from chain lengths, C6 to C22. Individual fatty acid levels are expressed as the percentage of total fatty acid of the formula used. The n6:n3 ratio was calculated by dividing the amount of all omega-6 polyunsaturated fatty acids (PUFAs) by the sum of all omega-3 PUFAs in each sample.

### Statistical analysis

The sample size for this study was based on prior studies in this model. The Shapiro-Wilk test was used to assess data distribution. Outcomes were assessed using analysis of variance (ANOVA), two-sample t-test, or log-rank test where applicable, and described as the mean ± the standard error of the mean (SEM) or 95% confidence interval (CI) where appropriate. We chose not to adjust for multiple comparisons due to limited sample size. Tests of significance were two-sided with P < 0.05 deemed statistically significant. GraphPad Prism (v10.3.1) software was used for analysis and generation of plots (GraphPad, Boston, MA).

## Results

ILC-mediated fat digestion was assessed under the conditions in the study using a colorimetric assay for free fatty detection. The average percentage of fat digestion in the formula was 66% (range 60–71%) for three separate ILC prototype lots (S1 Fig). As expected, there was no effective hydrolysis using the placebo cartridges (S1 Fig). The formulas for groups 2–4 were otherwise identical. Fat concentration of the enteral formula used in this investigation, 2:1 by-volume Similac Advance OptiGro and Esbilac Puppy Milk Replacer, was determined to be 45.54 mg/mL. Total fat concentration of each formula sample was analyzed to be 44.01 + 3.04 mg/ml. No major difference was seen between the measured levels of the omega-3 fatty acids alpha-linolenic acid (ALA), docosahexaenoic acid (DHA) and eicosapentaenoic acid (EPA), or the omega-6 fatty acids linoleic acid (LA) or arachidonic acid (ARA) between all sampled formulas (Table 1).

**Table 1:** Comparison of fat composition of unprocessed formula and formula processed by ILC and placebo cartridges. Fatty acid levels are expressed as the percentage of total fatty acid present in the formula sample. DHA – docosahexaenoic acid; EPA – eicosapentaenoic acid; ALA – alpha-linoleic acid; ARA – arachidonic acid; LA – linoleic acid.

Group	Docosahexaenoic Acid (DHA)	Eicosapentaenoic Acid (EPA)	Alpha-Linoleic Acid (ALA)	Arachidonic Acid (ARA)	Linoleic Acid (LA)	Ratio of Omega 6:3 Fatty Acids	Total Fat Content (mg/mL)
Formula	0.13%	0.03%	2.73%	0.22%	25.25%	8.81	45.97
Formula + Syringe/tubing	0.13%	0.03%	2.75%	0.21%	25.23%	8.74	42.83
Formula + ILC	0.14%	0.03%	2.76%	0.22%	25.46%	8.77	48.35
Formula + Placebo (Without Beads)	0.13%	0.03%	2.75%	0.22%	25.25%	8.76	41.31
Formula + Placebo (With Beads	0.13%	0.03%	2.76%	0.22%	25.27%	8.74	41.62

Normal controls (n = 24), housed with the dam and not subject to NEC induction, had a 100% survival rate on P7 (Fig 2C). The formula + ILC group (n = 37) had similar survival to the formula + placebo group (n = 41) on log-rank test (54.05 vs 46.34%, P = 0.57). The NEC + ILC cohort (n = 52) had similar survival to the NEC + placebo group (n = 65) (36.54 vs 21.54%, P = 0.14) (Fig 2C). All groups had similar starting weights on D1, and the weight trends over time are illustrated in Fig 2D. Normal controls experienced steady weight gain from baseline on D1 to end-of-experiment on D4 (1.071 ± 0.128 g). The formula + ILC group on average weighed 0.21 g more than the formula + placebo group on P7 (95% CI [−0.09, 0.52], P = 0.17), although this did not reach statistical significance. The NEC + ILC group weighed 0.34 g greater than the NEC + placebo group on D3 (95% CI [0.04, 0.64], P = 0.03); however, this did not reach statistical significance on D4 (P = 0.08). Inter-rater reliability was excellent, with intraclass correlation for clinical sickness and macroscopic gut scores of 0.91 (95% CI [0.87, 0.94]) and 0.92 (95% CI [0.87, 0.95]), respectively.

Clinical sickness and macroscopic gut scores are represented in Fig 3A and 3B, respectively. Clinical sickness, assessed from 0 to 12 in increasing severity, was lowest in the normal controls at 0.3 ± 0.2. The formula + placebo group had higher clinical sickness scores than the formula + ILC group (4.5 ± 0.3 vs 3.2 ± 0.4, P = 0.009). The NEC + ILC group had lower clinical sickness score than the NEC + placebo group (4.5 ± 0.4 vs 6.7 ± 0.6, P = 0.005). The clinical sickness score of the NEC + ILC group was not statistically different from that of the formula + placebo (P = 0.995). However, the clinical sickness score of the NEC + ILC group was higher than the formula + ILC group (4.5 ± 0.4 vs 3.2 ± 0.4, P = 0.02). Macroscopic gut scores, assessed from 0 to 6 in increasing severity, were lowest in the normal controls at 0.4 ± 0.2. The formula + placebo and formula + ILC groups had similar gut scores (3.2 ± 0.1 vs 2.9 ± 0.2, P = 0.37). The NEC + ILC group had lower gut scores than the NEC + placebo group (3.1 ± 0.2 vs 4.2 ± 0.3, P = 0.005). The NEC + ILC group additionally had similar gut scores to the formula + placebo (P = 0.67) and formula + ILC (P = 0.68) groups.

**Fig 4:**
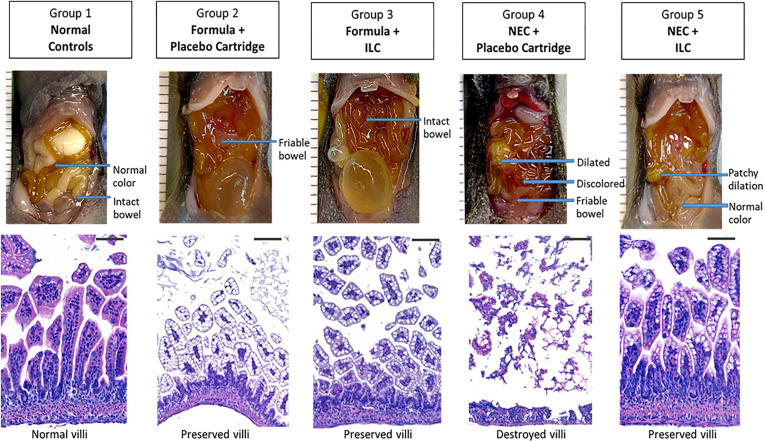
Representative gross and histological (hematoxylin/eosin-stained terminal ileum) specimens in a murine model of necrotizing enterocolitis (NEC). Group 1 – normal dam-reared controls. Group 2 – formula processed through the placebo cartridge. Gorup 3 – formula processed through the immobilized lipase cartridge (ILC). Group 4 – formula processed through the placebo cartridge under NEC induction. Group 5 – formula processed through the ILC under NEC induction. Bar indicates 50 µM.

Representative gross and histologic intestinal specimens are presented in Fig 4. Normal controls had intact intestines with normal coloring and villus architecture, remarked as having “no significant findings” per the masked independent pathologist. The formula + placebo and formula + ILC groups had intact intestine with rare segmental friability; these groups had preserved villi with “minimal inflammation and edema” per the pathologist. The NEC + placebo group had discolored, friable bowel with multiple dilated segments. On evaluation of terminal ileum histology, the NEC + placebo group had effaced villi with “moderate to marked inflammation” and “extensive intestinal erosions” per the pathologist. The NEC + ILC group had intact bowel with patchy areas of dilation; the villus architecture, however, was preserved throughout and the degree of inflammation was graded as “mild” by the pathologist.

The histologic severity of NEC was quantified from 0 to 4, in order of increasing severity, according to published protocol by the masked pathologist (Fig 3C) [19]. The normal controls had the lowest histologic severity scores with an average score of 0. The formula + placebo and formula + ILC groups had average scores of 0.4 ± 0.2. The NEC + placebo group scored higher than the NEC + ILC group (3.0 ± 0.8 vs 0.0 ± 0.0, P = 0.005) in histologic severity. The NEC + ILC group had similar histologic severity scores compared to the formula + placebo (P = 0.14) and formula + ILC (P = 0.14) groups.

## Discussion

In this study, we evaluated the impact of lipase-mediated external fat digestion in infant formula on disease severity of a neonatal murine model of NEC. Our findings demonstrate that the administration of ILC-digested enteral formula improved clinical, macroscopic, and histologic outcomes in NEC mice compared to placebo controls. Previously, Sodhi et al. demonstrated that the administration of “predigested” or “very low fat” formula reduced intestinal injury compared to “standard” fat in a similar murine model of NEC [16]. In this 2018 study, neonatal pups treated with chemically hydrolyzed fat had less oxidative damage and improved intestinal integrity compared to those given undigested fat. Thus, the investigators proposed that the accumulation of undigested fat within the intestinal lumen can trigger inflammation and mucosal damage, thus contributing to the development of NEC. An important limitation of the 2018 study was that the formulas not only differed in fat content, but also in the macronutrient composition. Thus, it is unclear whether their findings can be entirely attributable to the differences in formula fat digestion. In our current study, we used the same identical formula between groups with the only difference being related to the presence or absence of lipase-mediated fat digestion. Therefore, any differences between groups are related to the exogenous action of the immobilized lipase cartridge.

Nutrient absorption takes place in the villi. The relative intestinal surface area, the villus surface area divided by the total body surface area, is preserved between mice and humans, making the murine model suitable for dietary studies [20]. Several rodent models of NEC exist [17]. Experimental induction of NEC may involve a combination of exposure to commercial formula, bacterial toxins, fecal slurry, chemical detergents such as dextran sodium sulfate, hypoxia, and cold stress [21–24]. We used a modified version of a murine NEC model developed by Nolan et al., whereby we induced experimental NEC in neonatal pups with formula feeding, bacterial LPS, and hypoxia over a three-day experiment [23]. This was sufficient to induce NEC as validated by a masked pathologist (**Fig 4**) and avoids the variability that may be introduced from obtaining patient or other bacterial samples to induce NEC.

Mice in the NEC + placebo groups had worse survival than normal dam-fed controls (Fig 2C) and additionally had clear evidence of histologic injury (Fig 4). Survival outcomes were similar between NEC + ILC and NEC + placebo groups. This may be attributed to the limited sample size, or relatively short duration of the NEC model. However, the NEC + ILC group had improved clinical sickness, macroscopic gut, and histologic severity scores compared to the NEC + placebo group (Fig 3). Importantly, improved clinical sickness scores were also observed for the formula + ILC group compared to the formula + placebo group, suggesting some beneficial effect of the ILC without the NEC conditions.

Weight trends were overall similar between the formula-fed treatment groups (Fig 2D). Normal dam-fed controls exhibited steady weight gain over time, while formula-fed and NEC mice did not gain weight consistently. On average, the NEC + ILC group weighed 0.34 g more than the NEC + placebo mice on D3, which may signal enhanced nutrient absorption through the gut given that baseline weights were similar on P4. This is consistent with the gross and histologic findings, which demonstrated reduced NEC severity in the NEC + ILC group compared to the NEC + placebo group. Importantly, NEC severity scores of the NEC + ILC group were similar to those of the formula-fed, non-NEC mice which provides further evidence of the beneficial effects of ILC-mediated fat digestion on intestinal injury in this model.

Dietary fats play important roles in mediating gut health. Alshaikh et al. previously summarized the recent literature on the role of fat composition in the development of NEC specifically [25]. One study found that the supplementation of long chain polyunsaturated fatty acids (PUFA), specifically arachidonic and docosahexaenoic acid (DHA), attenuates intestinal injury in a neonatal rat model of NEC [26]. However, the administration of DHA alone (without arachidonic acid) may actually increase the risk of NEC according to a recent meta-analysis [27]. Notably, a balanced ratio of n-6 and n-3 PUFAs may be important in reducing intestinal inflammation as demonstrated in a colitis model [28]. Another murine NEC study demonstrated that short-chain fatty acid (SCFA) supplementation inhibited pro-inflammatory cytokine expression and reduced histologic gut injury [29]. The beneficial effects of SCFAs were similarly noted in a human study, in which infants with NEC were found to have significantly lower levels of total SCFAs compared to controls [30]. Analysis of the formula used in this study in its baseline, placebo cartridge-processed formula and ILC-digested formula revealed no major difference in omega-3 or omega-6 fatty acid composition, highlighting that the ILC cartridge preserves essential fatty acid concentrations and any difference seen in NEC severity may be attributed to triglyceride hydrolysis. These studies, taken together with our present findings, suggest that the pre-digestion of fats in enteral formula may reduce the risk of human NEC.

While there are well-established risk factors for NEC (e.g., prematurity), there is an ongoing effort to identify biomarkers that may be able to predict NEC prior to clinical onset. Low serum bicarbonate, low platelets, and high base deficits have been associated with increased risk for surgical NEC; specifically, a one-point decrease in bicarbonate was associated with 14% increased odds for NEC [31]. Additionally, a prospective cohort study involving 9 neonates with NEC demonstrated that elevation in serum intestinal fatty acid binding protein (I-FABP) levels was associated with NEC [32]. Although limited in enrollment, this study demonstrated that neonates who developed NEC while undergoing advancements in enteral feeding had a significantly higher level of serum I-FABP compared to controls, further highlighting the association between digestive burden and NEC. Of particular relevance to the present study is the identification of a single maternal breastmilk oligosaccharide, specifically disialyllacto-N-tetraose (DSLNT), and its association with NEC. In a prospective cohort of 33 infants with NEC, Masi et al. demonstrated that DSLNT levels were lower in breastmilk received by infants with NEC compared with healthy controls; there were associated shifts in the gut microbiome in these patients [33]. Breastmilk oligosaccharides form an active area of investigation, although presently their benefits are unclear and there is no current consensus regarding its supplementation in premature neonates. Breastmilk feeding is generally recommended to all neonates and is an accepted preventive strategy against NEC. Despite this, however, some neonates who receive breastmilk go on to develop NEC. A recent systematic review by Faizan et al. evaluated over 200 studies on the subject of biomarkers in NEC, and expertly identified other notable markers such as fecal calprotectin [34]. Despite the existence of biomarkers and data on breastmilk, it remains difficult to predict with precision the neonates who will develop NEC.

There are currently no FDA-approved treatments for NEC. The management in neonates is often supportive and involves bowel rest, administration of antibiotics, and/or surgical resection of the affected intestinal segment(s) if clinically necessary [35]. There are few modifiable preventive strategies in reducing the risk for NEC, with the exception of breastmilk feeding, which is not universally accessible [36,37]. Our study demonstrated that ILC-mediated digestion of enteral formula reduced clinical and histologic severity in a murine NEC model. It should be noted that while some improvements were observed with the administration of ILC-digested formula, these effects were insufficient to affect survival outcomes in this particular animal model of NEC. However, despite the modest effects of the ILC, human NEC is complex with varying degrees of clinical severity, thus the ILC may offer a protective, preventive effect in certain cases. An advantage of the present study is that it involved a neonatal prototype of a digestive cartridge that has already received FDA clearance for enteral nutrition support. The prototype cartridge utilized in our study was specifically designed for use in infants, a population at risk for NEC. Additionally, the vast majority of existing animal research involving NEC do not report mortality rates. Our present study measured survival as an outcome; although there were no statistically significant differences in group-specific mortality rates, we demonstrated improvements in other clinical parameters with application of the ILC such as clinical sickness and histologic intestinal injury. Overall, our results contribute to the growing body of evidence that undigested fats can mediate intestinal injury. The ILC may be most beneficial when utilized as a preventive strategy against NEC, whereby neonates who are identified as high risk (e.g., prematurity, low bicarbonate, high or rising I-FABP) requiring enteral nutrition are fed ILC-digested formula capable of producing more easily absorbable forms of dietary fat.

### Limitations

There are several limitations to this study. The murine model of NEC does not replicate human NEC. Mice and humans have significant differences in intestinal physiology and immunity. This experiment involved induction of NEC in neonatal mice using artificial stressors in a controlled laboratory setting, whereas human NEC is complex and multifactorial. NEC animals did not receive typical maternal care. Proxy dams could not be provided due to physical aggression that adults exhibit towards non-related pups. To overcome this limitation, pups of the same treatment group were housed together and provided stimulation to one another. Investigators additionally provided manual stimulation several times per day in the form of weighing, orogastric gavage feeding, and transfers to and from the hypoxia chamber. Despite the use of littermate pups, mice inherently vary in physiology. Bias may be introduced by weaker mice succumbing to NEC induction prior to the experimental end point, which can limit the interpretation of the current findings. Cause of death of individual animals could not be identified, though no deaths were observed as a result of technical error (i.e., orogastric feeding). The sex and gut microbiome of the mice were not evaluated, thus any sex- or microbiota-specific effects on NEC severity are unknown. Additionally, potential mechanisms that may explain the current findings were not investigated. We attempted to conduct assays for markers of inflammation however, these assays resulted in levels below the detection limit and we were not able to draw meaningful conclusions. This may be related to the short half-life of cytokines. Unfortunately, the limited amount of tissue and blood samples prevented us from investigating additional cytokines. This data is important and would be of interest in future mechanistic investigations. We did not evaluate rodent or human breastmilk under NEC induction in this experiment, which may be considered for future experiments. Additionally, it should be noted that this experiment involved a preventive, not treatment, strategy for NEC. The administration of ILC-digested formula occurred concurrently with experimental NEC induction, not after the establishment of NEC. It is unclear whether ILC-mediated fat digestion would have any “rescue’ effects, and may be of interest for future studies. Despite these limitations, this study provides proof-of-concept data that lipase-mediated digestion of fat in enteral formula is a clinically feasible therapeutic target for reducing NEC burden.

## Conclusion

Feeding with commercially available formula is associated with an increased incidence of NEC, particularly among premature infants, who have underdeveloped gastrointestinal systems and relative lipase deficiency. Where breastmilk is unavailable, fats in enteral formula can be pre-digested to potentially reduce intestinal injury. In a murine model of NEC, administration of enteral formula pre-digested by the ILC improved clinical sickness scores, macroscopic gut scores, and histologic severity of NEC compared to formula processed through an inactive, placebo cartridge. These findings support further investigation of the ILC as a non-invasive, preventive therapy for NEC in humans with the potential to reduce the need for operative intervention.

### Statement on public access policy

The “Public Access Policy” of the National Institutes of Health mandates that Author Accepted Manuscripts arising from NIH-funded research must be made available in PubMed Central immediately upon publication. By accepting this manuscript, which arose from NIH-funded research, the Journal agrees that it will not impose a fee for the author#39;s compliance with the NIH Policy, notwithstanding any agreement or policy of the Journal to the contrary.

## Supporting information

S1 Fig Fat Hydrolysis of the immobilized lipase cartridge compared to placebo cartridges. Each experiment was performed in triplicate for data verification.
(DOCX)

## Abbreviations

ANOVA: analysis of variance
ARA: arachidonic acid
ALA: alpha linolenic acid
CDC: Centers for Disease Control and Prevention
DHA: docosahexaenoic acid
EPA: eicosapentaenoic acid
FDA: Food and Drug Administration
H&E: hematoxylin and eosin
IACUC: Institutional Animal Care and Use Committee
ILC: Immobilized lipase cartridge
LA: linoleic acid
LPS: lipopolysaccharide
NEC: necrotizing enterocolitis
NICU: neonatal intensive care unit
NIH: National Institutes of Health
P#: post-natal day #
SEM: standard error of the mean
