# Supplementary figures and images for: A digestive cartridge reduces intestinal injury in a murine model of necrotizing enterocolitis
Source: PLoS One. 2026 Apr 30;21(4):e0348200. doi: 10.1371/journal.pone.0348200 (PMC13132229; doi:10.1371/journal.pone.0348200)

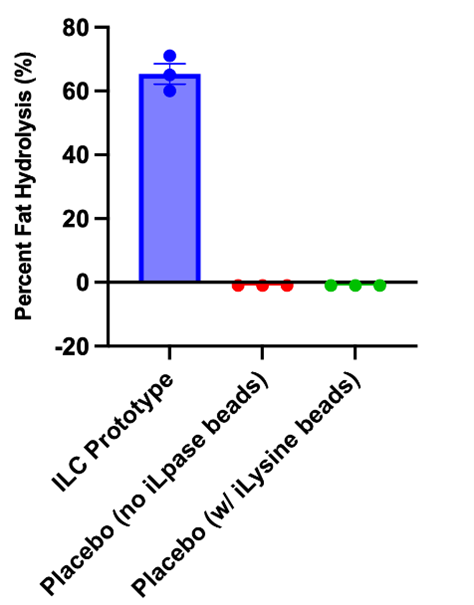


**S1 Fig.** Fat Hydrolysis of the immobilized lipase cartridge compared to placebo cartridges

Supplement: S1 Fig — Each experiment was performed in triplicate for data verification. (DOCX) [file pone.0348200.s001.docx]
